# Provider and Veteran Perspectives on the Resources Needed to Mitigate Veteran Homelessness

**DOI:** 10.1002/jcop.70093

**Published:** 2026-02-16

**Authors:** Lourence Misedah‐Robinson, Rebecca. L. Kinney, Jack Tsai

**Affiliations:** ^1^ Houston Center for Innovations in Quality, Safety, and Effectiveness (IQuESt), Michael E. DeBakey Veterans Affairs Medical Center Houston Texas USA; ^2^ VA National Center on Homelessness Among Veterans, U.S. Department of Veterans Affairs Washington, DC Washington USA; ^3^ Baylor College of Medicine Houston Texas USA; ^4^ Department of Population and Quantitative Health Sciences University of Massachusetts Medical School Worcester Massachusetts USA; ^5^ Department of Management, Policy, and Community Health The UT Health School of Public Health Houston Texas USA; ^6^ Department of Psychiatry Yale School of Medicine New Haven Connecticut USA

**Keywords:** affordable housing, community needs assessment, homeless service providers, homelessness, mental health, support services, veterans

## Abstract

We aimed to expand understanding of provider/Veteran attitudes about resources and services that would mitigate Veteran homelessness. We analyzed qualitative data from the 2024 Community Homelessness Assessment, Local Education, and Networking Groups for Veterans survey. In all, 4,990 respondents completed the survey: 2,928 (59%) homeless program service providers, clinicians, and staff and 2,062 (41%) homeless experienced Veterans. Qualitative analysis identified six key themes, which included: 1) increasing affordable, safe housing options; 2) expanding Housing and Urban Development‐Veterans Affairs Supportive Housing; 3) increasing access to mental health resources and services; 4) more external support and collaborations; 5) increasing financial support and resources; and 6) increasing employment and job training. Providers prioritized mental health services, while Veterans more frequently emphasized needs such as housing and financial support. Veteran homelessness is complex and needs integrated approaches to service delivery that address unique challenges and improve overall outcomes.

## 1 Introduction

Homelessness among Veterans remains a significant public health concern that highlights the complexities involved in supporting those who have honorably served our nation (Kinney et al. 2024b; Tsai and Rosenheck 2015). Homeless‐experienced Veterans (HEV) are U.S military Veterans who are currently or were formerly homeless. Although tremendous progress has been made in reducing homelessness in the past decade (De Sousa and Henry 2024; Tsai et al. 2021), homelessness among Veterans remains an important VA priority. Veteran homelessness is a complex and interrelated phenomenon (Allegrante and Sleet 2021; Sleet and Francescutti 2021) due to economic downturns, a scarcity of affordable housing, individual‐level factors, including mental health and substance use (Betancourt et al. 2023; Byrne et al. 2016; Dunne et al. 2015; Edwards et al. 2024; Tsai et al. 2025), numerous emergent community shortfalls associated with moving unsheltered HEV into transitional and/or permanent housing (U. S. Government Accountability Office 2023) and other known and unknown factors. To mitigate the homeless crisis, it is essential to reevaluate the current needs and interventions that engage HEV in VA healthcare and homeless services.

The Community Homelessness Assessment, Local Education, and Networking Groups for Veterans (Project CHALENG) was launched in 1994 in response to Public Law 102–405, which seeks to enhance the continuum of services for homeless Veterans provided by VA health care facilities and community service agencies. Annually, the CHALENG survey is distributed to Veterans and providers across various settings, including those in and outside the VA healthcare system. The results are used to identify existing gaps in services and resources needed to mitigate Veteran homelessness (U. S. Department of Veteran Affairs 2025b).

Although homelessness among Veterans has gradually decreased over the past decade, a larger percentage still experiences sheltered and unsheltered homelessness, highlighting the ongoing need for regular assessments (Diaz 2025; U.S. Department of Veteran Affairs 2025). Consistent with CHALENG data from 2023 and the previous 20 years, the 2024 findings show that unmet needs are primarily services that VA cannot provide directly. This underscores the importance of sustained collaboration between VA and community partners to address these gaps and prevent returns to homelessness. Therefore, the CHALENG survey offers the opportunity to examine evolving patterns and reinforce the importance of capturing both Veteran and provider perspectives.

Prior studies using the CHALENG survey have also underscored where critical resources are still needed for homeless and at‐risk Veterans, such as credit counseling, utility assistance, and dental care (Tsai et al. 2019). Additionally, studies have highlighted the important role of housing, finances, employment, and emotional support services in mitigating Veteran homelessness (Tsai et al. 2022). However, most prior studies using CHALENG data have relied solely on responses from homeless and at‐risk Veterans and have not examined them alongside responses from service providers who also participate in the CHALENG survey. Provider input is essential at both the national and local levels to inform legislative proposals, support new program development or expansion, guide strategic planning between VA medical centers and community partners, and demonstrate need in grant applications. Providers may also identify unmet needs differently than Veterans, offering complementary insight into service gaps and priorities. A comprehensive examination of both Veteran and provider perspectives on the resource needs of this population may further our knowledge in this area.

This study aimed to build on previous work by qualitatively examining providers' and Veterans' perceptions of the resources and services needed to end homelessness among Veterans. A Comprehensive examination of both perspectives may deepen understanding of resource needs and strengthen efforts to address Veteran homelessness.

## 2 Methods

We analyzed data from the 2024 CHALENG survey. Of specific interest was the open‐ended response format that queried respondents' recommendations on “the most important resource/service that could help end Veteran homelessness in your community”.

## 3 Recruitment of Participants

CHALENG is open to all Veterans, not limited to those receiving VA services. The full details surrounding the recruitment of CHALENG respondents are referenced elsewhere (Tsai et al. 2023). Briefly, Providers, staff, and HEV are recruited nationally and offered the opportunity to complete the survey in either a hard‐copy or online format (Tsai et al. 2023). The survey is distributed multiple times throughout the year at Stand Downs, VA homeless assessment centers, and homeless programs such as the Domiciliary Care for Homeless Veterans, Healthcare for Homeless Veterans, Grant and Per Diem, and HUD‐VASH. It is also disseminated at CHALENG meetings and other Veteran‐driven events nationwide. Hard copies are distributed at all VA homeless program sites, and the survey is anonymous. In addition, a website allows individuals to complete the survey online. Completed surveys are accumulated throughout the year from participating sites. The VA Homeless Program Office implements the CHALENG survey as part of quality improvement, with approval from the Office of Management and Budget.

## 4 Analysis

Analysis proceeded in two steps. First, analyses were performed using STATA (Stata Corporation LLC, College Station, TX) to produce descriptive sociodemographic statistics on survey respondents, including staff and providers' agency and location of employment, and Veteran demographic data. Second, data from the open‐ended question, which queried provider, staff, and Veterans about their perceptions of the “the most important resources and services that could help end Veteran homelessness in your community,” were analyzed qualitatively. Perspectives were examined across two groups: providers and staff versus the Veteran cohort. Two investigators (LM, RK) conducted open coding of the responses, creating code definitions and schemes inductively as concepts emerged from the data (Cordasco et al. 2022; Nowell and Albrecht 2019). To systematically compare perspectives, responses were coded independently by respondent type (provider/staff *vs.* Veterans), identifying shared and divergent themes across groups. The coders identified codes with similar meanings and merged and organized them into broader themes that covered the meanings. Both coders compared the codes, discussed them, and reached a consensus when there were discrepancies. Comparative analysis focused on the frequency, emphasis, and contextual framing of themes within each group. A third investigator (JT) was available to consult about any remaining discrepancies. Qualitative analyses used Altas. ti (Atlas. ti Scientific Development, GmbH).

## 5 Results

A total of 4,990 individuals responded to the CHALENG survey, of which 2,928 (59%) were service providers or staff and 2,062 (41%) were HEV. Table 1 displays the characteristics of the survey respondents. The majority of providers and staff respondents were employed by the VA and were delivering care at medical centers. Veteran respondents were more likely to be male, non‐Hispanic white, and residing in urban/suburban areas.

**Table 1:** Characteristics of CHALENG Survey Respondents.

Characteristics	Provider/staff (*N*=2928)	Veterans (*N*=2062)
Current agency of association		
VA	1,528 (52%)	—
State/local government	843 (29%)	—
Private, nonprofit	499 (17%)	—
Other federal agency	58 (2%)	—
VA position		
*VA Medical Center*	1,085(71%)	—
*Community‐based outpatient clinic*	351(23%)	
*VA Central Office*	31(2%)	
*Veterans Integrated Service Network (VISN)*	61(4%)	
Gender	—	
Male		1855(90%)
Female		207(10%)
Race	—	
White		1092 (53%)
Black, African American		660 (32%)
American Indian, Native American		206 (10%)
Asian		20 (1%)
Missing		84 (4%)
Ethnicity	—	
Non‐Hispanic, Latino/a		1876 (91%)
Latino/a		186 (9%)
Current Living Situation	—	
Literally homeless		379 (19%)
Permanent subsidized housing		630 (31%)
Transitional housing		533 (26%)
Unsubsidized housing		383(19%)
Emergency housing		87 (4%)
Missing		50 (1%)
Location	—	
Urban/suburban		1608(78%)
Rural		454(22%)
Experienced four or more episodes of homelessness in the past three years	—	784(38%)
Health status (self‐reported)	—	
Poor		680(33%)
Fair/good		1052(51%)
Very good/excellent		309 (15%)
Missing		21(1%)

The qualitative analysis of responses to “the most important resources/services that could help end Veteran homelessness in your community” identified six predominant themes, which included: 1) increasing affordable, safe housing options; 2) expanding Housing and Urban Development Veterans Affairs Supportive Housing (HUD‐VASH); 3) increasing access to mental health resources and services; 4) increasing external support and collaborations; 5) increasing financial support and resources; and 6) increasing employment and job training. Each theme, along with its subthemes, is discussed in detail below. Table 2 shows the frequency with which providers mentioned each priority compared with the HEV cohort.

**Table 2:** Comparison of Most Frequently Identified Needs between Provider and HEV Cohort.

Providers			Veterans		
Theme	*N*	%	Theme	*N*	%
Access to housing	1140	40%	Access to housing	736	55%
Mental health	409	14%	HUD‐VASH expansion	292	22%
HUD‐VASH expansion	327	11%	External support	160	12%
External support	253	9%	Financial support, resources	150	11%
Financial support, resources	178	6%	Employment, job training	146	11%
Outreach	128	4%	Financial services	77	6%
Education, life skills	125	4%	VJII	50	4%
Employment, job training	125	4%	Access to healthcare	21	2%
Access to healthcare	117	4%	Mental health	20	1%
VJI	84	3%	Education, life skills	13	1%
Financial services	40	1%	Outreach	11	1%

Abbreviations: HEV, homeless experiences Veterans; HUD‐VASH, Housing and Urban Development‐Veterans Affairs Supportive Housing; VJI, veteran justice involved.

## 6 Theme 1: Access to Affordable, Safe Housing

Affordable, safe housing was the number one needed resource to end Veteran homelessness reported across both provider and Veteran respondents. Providers and HEV varied in their perceptions of what access to affordable, safe housing entailed among different marginalized groups.

HEV emphasized that affordable, permanent housing provides a stable foundation for Veterans to overcome other life challenges associated with homelessness. One shared:

I have not lived in an apartment since 2016. I have slept in dirty motels and am currently sleeping in my car. I slept the entire winter of 2021 in my car and have previously slept the entire summer. Having a nice, safe place to sleep is most important.

(Veteran, Wisconsin)

Tiny house communities were an option mentioned by both providers and HEV. Respondents envisioned these communities as private and supportive environments that could foster stability and community.

Homeless Veterans want our help. I thought building tiny houses on acres of land just for homeless Veterans would help take in more…[so] why not buy 50 acres of land, build tiny houses just for homeless Veterans, and help them get back on their feet again.

(Provider, VA Medical Center, Tennessee)

One respondent noted that tiny house communities can often accommodate Veterans with pets, which can be a barrier to securing housing. They explained, “ Tiny homes can offer Veterans their own outdoor area and where they can have a pet. These homes would be furnished and come with a basic housing kit, such as cleaning supplies, kitchen items, and a washer and dryer.” (Provider, VISN, Arizona)

### 6.1 Housing to Accommodate the Elderly and Disabled

Both Veterans and providers frequently commented on the need for housing resources for disabled and aging individuals who could not manage their activities of daily living. Veterans emphasized the importance of in‐home support services and assistance with daily tasks in accessing stable housing, independence, and mitigating returns to homelessness among disabled and aging Veterans. One illustrated how a sudden disability led to housing loss and extreme living conditions, reinforcing the need for tailored housing and support services:

I broke my leg in half in three places, and I could not work. Once my savings were gone, I was kicked out of my apartment and had to find a place to sleep. I found an old boat in the woods, so I crawled inside and lived there for about 4 months. There was no running water, electricity, or even a screen to keep bugs out.

(Veteran, Tennessee)

Providers also recognized the need for housing options for elderly people and people with disabilities. One provider described specific needs, “In‐home cleaning services and assistance with activities of daily living to keep the elderly out of nursing homes” (VA Medical Center, Pennsylvania). Another staff member went on to emphasize, *“*There is a need for housing for Veterans who need healthcare services and are aging but not ready yet for nursing homes and don't have the money for assisted living*”* (Provider, VA Outpatient Clinic, Kentucky). Collectively, both Veterans and providers agreed that there is a need for “better resources to support aging Veterans who can no longer live independently” (Provider, VA Medical Center, California).

### 6.2 Emergency Housing

Providers reported that emergency housing for Veterans at immediate risk of harm or displacement was a prioritized needed resource, while Veterans did not consistently identify it as a primary need. One provider noted, “Understanding root causes and ensuring rapid housing for those experiencing domestic violence and not having income be a barrier since that can often be tied up in violent relationships” (Provider, VA Medical Center, Iowa). Another provider added, “I work with those who have experienced violence, and there is a need for easy access to housing on an emergency level with little criteria*”* (Provider, VA Medical Center, Wisconsin).

### 6.3 Veteran Justice‐Involved Housing

A final subtheme was the need for Veteran Justice‐Involved (VJI) to have affordable, safe housing options. Both Veterans and providers reported that many landlords and programs were reluctant to accept individuals with felony records, which significantly reduces available housing options for these Veterans. One respondent noted, “There are limited housing options for Veterans who are felons upon their release from prison, specifically, those who have been incarcerated for sexual offenses” (Provider, Mental Health Department, West Virginia). These Veterans are often subjected to geographical residency restrictions in their efforts to secure housing, given that they have been placed on sex offender registries. Another agreed that “It is incredibly challenging to find housing for Veterans with residency restrictions due to being on a sex offender registry” (Provider, VA Medical Center, Kentucky).

## 7 Theme 2: **HUD‐VASH Expansion**

### 7.1 Expand Eligibility

Both provider and HEV respondents frequently cited the HUD‐VASH program as a critical resource for addressing homelessness. Respondents expressed concerns about its limitations and called for expanded eligibility. One underscored the need to expand the model to Veterans who may not qualify.

I think wrap‐around case management services are so important. I see them being successful in the HUD‐VASH program and would love to see more of that higher‐intensity case management for folks dealing with homelessness who are NOT eligible for HUD‐VASH.

(Provider, VA Medical Center, Wisconsin).

HEV with dishonorable discharge face additional housing barriers, as some may be ineligible for HUD‐VASH. One Veteran recommended “Removing any barriers, restrictions, income levels, etc., on housing assistance qualifications since not everyone's situation is the same” (Veteran, Georgia). Providers agreed that HUD‐VASH eligibility should be expanded to all Veteran groups. One shared, “Clearer resources are needed for Veterans with less than honorable or dishonorable discharge status” (Provider, private nonprofit community‐based organization, Virginia).

### 7.2 More Vouchers

In addition to expanding eligibility, many providers called for an increase in HUD‐VASH vouchers. One described the economic challenges:

HUD‐VASH programs and support funds to assist with the gaps in the cost of fees and the difference in the cost of housing due to inflation. The cost of housing and the amount of housing vouchers allowed must be examined to see the challenges faced by homeless Veterans.

(Provider, VA Medical Center, Louisiana)

Veterans concurred, noting that, even when an individual qualifies, the scarcity of vouchers and the rising cost of housing are barriers. They described how HUD‐VASH vouchers transformed their lives, while also noting delays and limitations in the process. One stated, “My HUD‐VASH voucher will help me get housing, but I am waiting on inspection…if I did not have this voucher, I would not have housing” (Veteran, Kentucky).

### 7.3 Case Management

Providers prioritized expanding HUD‐VASH case‐management services, highlighting the essential role of case managers in helping Veterans navigate complex systems and maintain housing stability. One provider stressed that high caseloads limit case managers' ability to provide effective support in helping Veterans obtain and sustain permanent housing, “More case managers are needed to help reduce caseloads so that effective services, linkages, and/or referral management can occur. Twenty‐five cases to one person working in homelessness is not appropriate for the homeless population” (Provider, VA outpatient clinic, California). Another provider highlighted the role of case managers in bridging gaps between providers and systems, noting, “I see how HUD‐VASH case managers really help Veterans get connected and navigate the gaps between MD, medical, etc.” (Provider, VA Medical Center, Wisconsin).

## 8 Theme 3: Access to Mental Health Resources and Services

Providers reported the need for accessible and comprehensive mental health services. In contrast, Veterans prioritized mental health as less of a need but shared some personal experiences that underscored the detrimental effects of untreated mental health conditions on housing stability.

### 8.1 General Mental Health

Providers frequently emphasized general mental health needs, including the necessity for improved access to mental health and substance use disorder treatment services. One described the need for “more mental health resources for Veterans with physical disability/impairments to be able to transition to after a skilled rehab stay” (Provider, VA Medical Center, Tennessee). Veterans also recognized the intersection of mental health and housing stability. One shared, “I need to talk to mental health specialists because I like to ‘run away’ a lot, and I also have trouble with impulse…so I have found myself addicted to drugs and homeless many times” (Veteran, Florida).

### 8.2 Suicide Prevention Resources

Providers also underscored the need for suicide prevention resources. One stated, “There is also a huge need for treatment centers for the homeless who struggle with addiction…which can lead to suicide. We need to focus more on helping them get back on their feet and suicide prevention*”* (Provider, private nonprofit community‐based organization, North Dakota).

### 8.3 Substance Use Disorder Treatment

Providers also emphasized the need to integrate housing and mental health/substance use recovery services, suggesting more collaborations between VA clinical recovery programs and housing services. One provider stated, “After permanent housing”, we need to focus on a level of accountability about sobriety. Having a Veteran enroll in a 12‐step program, abstinence program, harm reduction, etc., keeps the Veteran involved and accountable while receiving housing assistance (Provider, Private Non‐profit Community‐based Organization, California). Another provider highlighted a gap in treatment access, noting, “We have a lot of SUD treatment beds at The Healing Place, but they do not allow suboxone or SUBLOCADE”. This is a major barrier to housing Veterans (Provider, VA Outpatient Clinic, Kentucky).

## 9 Theme 4: External Support

Providers and HEV agreed on the need for additional external supports and resources to mitigate homelessness. Providers noted that structural supports such as transportation and partnerships were warranted, while HEV highlighted the need for comprehensive services and legal aid to navigate the complexities of homelessness and reentry.

### 9.1 Public Transportation

Providers cited transportation as the most needed external support. They described how transportation insecurity can lead to a cascade of adverse outcomes. One explained:

I have spoken with many Veterans who have spiraling issues due to transportation insecurity that can lead to homelessness. For instance, Veterans who have major vehicle issues, which lead to loss of jobs, costly loans taken out for repairs, and eventual eviction dangers.

(Provider, VA Medical Center, Arizona)

### 9.2 Local Partnership and Support

Providers emphasized the need for more local partnerships, advocating for collaborations among the VA, local governments, and nonprofit organizations to create more comprehensive support systems. They noted that partnerships between the VA and local government agencies could bridge service gaps, particularly for HEV who are ineligible for VA care. One advocated for community‐based alternatives, stating “The VA should partner with state, county, and municipal governments to create small house‐type communities for these Vets” (Provider, VA outpatient clinic, Tennessee). Providers also reported the need for community‐based alternatives, particularly for HEV who ineligible for VA care and benefits are, “Some Vets do not have access to or qualify for VA care. There needs to be an alternate small building on site where nonprofits can come in and provide care for Veterans who are not eligible.” (Provider, VA outpatient clinic, Tennessee). These partnerships were recognized as a needed resource for “Obtaining state‐level or community support to improve housing for Veterans” (Veteran, Tennessee)

### 9.3 Comprehensive Services

Veterans mentioned the need for comprehensive services, including coordinated access to mental health care, substance use treatment, housing, and legal support. One stated, “Accessing Eagle Star has been a huge benefit. From there, HUD/VASH contacted me…These services were needed to get me off the street and into permanent housing” (Veteran, New York). Another expressed that there is a need for “referrals to community organizations that are willing to work with a Veteran with a recent eviction” (Veteran, Pennsylvania).

Providers echoed the need for streamlined access to services. One stated the need to simplify and expedite housing services, “Connect Veterans to housing options quickly (i.e., with minimal barriers or waiting periods” (Provider, VA Medical Center, California). Another added:

I have heard from many Veterans that they have to wait a long time to receive the care and services they need. I think we need to look into shortening these wait times. In extreme cases, families have said that the Veteran passed before receiving care. We can do better.

(Provider, VA Medical Center, Illinois).

## 10 Theme 5: Financial Support and Resources

A fifth theme, frequently identified by HEV, was the need for financial support. Veterans frequently mentioned income stability, disability benefits, and financial literacy as essential to achieving and maintaining housing, while providers focused on structured financial assistance such as cash aid.

### 10.1 Income Support

Veterans frequently mentioned income as the most critical financial resource. One stated, “Give financial assistance when needed” (Veteran, Nevada). Another described the strain of limited income on family well‐being, “Low‐interest house loans, immediate needs for cash. If VA wants me to live on a monthly payment, I can do that, but do not tell me to make my wife suffer” (Veteran, Nevada). Some also highlighted the importance of Social Security Income and disability benefits. Another advocated for “continuance of Social Security benefits with annual increases equal to inflationary rates is needed” (Veteran, New York).

### 10.2 Financial Education and Credit Repair

Veterans reported the need for financial literacy to prevent housing instability. One stated the need for “financial management combined with constructive activities, continued education, [and] exercise” (Veteran, Pennsylvania). Providers concurred, “Some Veterans make enough money, but their mental health issues or problems make them manage their finances poorly and cause them to be homeless” (Provider, VA Medical Center, Wisconsin).

## 11 Theme 6: Employment, Job Training

Veterans underscored a sixth theme, Employment and Job Training, which included the importance of financial education, job placement, and career development for achieving long‐term housing stability.

### 11.1 Financial Education

Veterans stressed the need for greater financial literacy and job training to mitigate Veteran homelessness. They often emphasized the need for “financial management combined with constructive activities, continued education and exercise” (Veteran, Pennsylvania), while providers reported the need for more structured interventions, such as more organized concrete, consistent living skills, mandated financial education/budgeting once housed (Provider, VA Outpatient Clinic, California). Another proposed

A program that could allow the Veteran who cannot manage their money to pay the landlord directly, so if the Veteran has responsibility issues, they would not be kicked out for nonpayment. Some Veterans make enough money, but their mental health issues or problems make them manage their finances poorly and cause them to become homeless.

(Provider, VISN, Wisconsin)

### 11.2 Job Seeking and Placement

Both Veteran and provider respondents emphasized the need for structured support during the transition from military to civilian life. One noted, “Leaving the military is traumatic. Job training and mental health therapy appointments for the first year coming out of the military would go a long way” (Provider, private nonprofit community‐based organization, Texas).

Other providers indicated that more tailored employment programs were needed, A program, sort of like the Job Corps program…where the Veteran could feel more empowered and capable (Provider, VA Medical Center, Tennessee). A Veteran also shared the experience of struggling with job seeking and placement, noting that often employment was insufficient to cover living expenses, “The part‐time employment that I did have was not livable…more dedicated employment assistance or education counseling before and after a Veteran leaves the program is needed” (Veteran, Tennessee).

## 12 Discussion

Despite the overall strides that the VA has made in reducing Veteran homelessness, this study highlights some specific resources and service gaps mentioned by providers and Veterans in efforts to mitigate Veteran homelessness. Notably, this study focused on synthesizing the perspectives of providers and Veterans on their needs. Certainly, there are important contextual and financial constraints that need to be considered that may not have been factored in.

Qualitative data from homeless providers/staff and HEV identified six themes, including access to affordable, safe housing; HUD‐VASH expansion; mental health resources and services; external support; financial support resources; and employment and job training.

Access to affordable, safe, permanent housing has been a VA priority in mitigating Veteran homelessness. However, providers and HEV emphasized that the lack of affordable, safe housing remains a persistent barrier for underserved populations such as justice‐involved, disabled, elderly, and rural Veterans. These findings echo decades of CHALENG and national VA data showing that structural inequities and geographic disparities continue to limit sustainable housing access, even amid overall declines in homelessness (Cusack et al. 2022; Kinney et al. 2024a; O'Toole et al. 2024). Several federal initiatives have attempted to respond to these needs, including the HUD‐VASH program (2024), which expanded eligibility by excluding VA service‐connected disability payments from income calculations and raising the income threshold to 80% of the Area Median Income (U.S. Department of Housing and Urban Development 2024). VA also continues to provide Specially Adapted Housing and Special Home Adaptation grants to assist Veterans with service‐connected disabilities (U. S. Department of Veteran Affairs 2025a). However, some Veterans still lack access to affordable, safe housing. Consistent with conceptual frameworks, such as the Journey to Home conceptual model (Cusack et al. 2020), which seeks to understand the risk factors for homelessness among HEV and trace their paths through housing instability, research also indicates that intersecting vulnerabilities, such as justice involvement, can worsen housing instability (Holliday et al. 2022; Holliday et al. 2022). This underscores the importance of life‐course, trauma‐informed approaches that support Veterans during their homeless experiences. Future efforts should focus on targeted interventions for high‐risk populations, improving rural housing infrastructure, and evaluating the sustainability of housing placements. It is also essential to strengthen landlord engagement, offer incentives, and create integrated care models that address housing and health needs while ensuring ongoing evaluation of these programs across diverse Veteran groups.

Our analysis found provider responses consistent with the previous literature that emphasizes the role of behavioral health in housing stability. Previous CHALENG data and VA research have demonstrated that clinical interventions, such as psychiatric care and substance use treatment, are essential components of housing stability (Tsai et al. 2019; Tsai et al. 2022). Still a divergence exists and may stem from a disconnect between clinical frameworks and lived experience. Providers trained in behavioral health and systems‐level care may view mental health as the root cause of homelessness. Veterans, however, might not fully recognize or acknowledge their mental health conditions or may not perceive them as directly linked to their housing instability, particularly when basic needs are unmet or when mental illness is normalized within their social context (Tsai et al. 2025). This gap in insight might contribute to differing priorities between HEV and providers and underscores the importance of trauma‐informed, Veteran‐centered approaches that integrate housing with accessible, nonstigmatizing mental health care. Trauma‐informed approaches that emphasize safety, empowerment, and relational trust can help mitigate avoidance and disengagement among Veterans who have experienced institutional betrayal, reframing engagement as a partnership rather than compliance (Fuseini et al. 2022; Kelly et al. 2014).

Providers may also identify unmet needs differently than Veterans. For example, housing for registered sex offenders is consistently described by providers as a high‐priority unmet need. In the 2023 CHALENG cycle, it was the second‐most frequently mentioned unmet need among both Veterans and providers, continuing a decade‐long trend (U. S. Department of Veterans Affairs 2025). Previous studies have also shown that Veterans with sex offense histories face disproportionately higher risks of housing instability and homelessness compared to other Veterans. (Byrne et al. 2022). These challenges are compounded by exclusion from housing programs such as HUD‐VASH (Finlay et al. 2019; Simmons 2018) and by systemic barriers that persist even when Veterans are eligible for services (Kelton et al. 2025). These insights highlight the value of provider input in addressing complex, persistent gaps in housing access. Strengthening coordination between VA and community partners can help translate these insights into more equitable housing solutions for underserved Veterans.

Both providers and HEV support expanding the HUD‐VASH program. In 2024, the VA reaffirmed this commitment by promoting and advancing the availability of affordable housing for HUD‐VASH project‐based voucher development and targeted availability of rental subsidies (U. S. Department of Veteran Affairs 2024a). This approach to the lack of affordable housing for homeless Veterans includes enhancing residential homeless programs, including the Grants and Per Diem program, which funds nonprofits that help unhoused Veterans get into transitional housing and eventually find permanent homes. Currently, the VA is implementing a continuous evaluation of current policies, which will identify and remove barriers to housing and ensure that programs operate in an equitable manner across race, sex, sexual identity, socioeconomic status, and legal history disparities (U. S. Department of Veteran Affairs 2021). For example, the HUD‐VASH and Supportive Services for Veteran Families programs are collaborating to increase voucher use and accelerate housing placements among Veterans with families (U. S. Department of Veteran Affairs 2024a). The benefits of secure, affordable housing can be observed across several dimensions, including improving medication adherence, decreasing bias from care providers, providing a community that promotes social support systems, and allowing Veterans to devote financial resources to other basic or preventive needs (Crone et al. 2022). Still, investing in ongoing funding and research that targets underserved HEV populations is warranted. Integrated wraparound models that co‐locate mental health care, social services, and peer support have been shown to improve long‐term housing retention compared with segmented programs (Tsai and Rosenheck 2015).

Veterans emphasized the need for direct financial support, including income assistance, access to Social Security Supplemental Income or Social Security Disability Insurance, and financial literacy. Recovery‐Oriented Money Management is one intervention that has demonstrated promise in enhancing HEV's financial decision‐making and mitigating housing instability(Tsai et al. 2019). There is an ongoing need for financial literacy and empowerment programs that go beyond budgeting to include credit repair, debt management, and understanding of VA and non‐VA benefits (Katie E Davenport et al. 2023). Financial instability remains both a precursor to and a consequence of homelessness. Structured money management and financial education programs, when integrated into VA case management, have been shown to reduce relapse and support long‐term housing stability (Elbogen et al. 2013; Nelson et al. 2021). Programs that combine financial education with peer support and case management have demonstrated effectiveness in promoting long‐term housing stability and economic independence (Katie E. Davenport et al. 2023; Elbogen et al. 2016).

VA's homeless programs serve hundreds of thousands of homeless and at‐risk Veterans each year. VA homeless and medical services that are co‐located in the same physical space are effective in providing HEV with housing solutions, employment opportunities, health care, justice, and reentry‐related services (O'Toole et al. 2016). Yet there still remains an access barrier for HEVs who are ineligible for VA care or may not have access to health services that match their medical needs (Crone et al. 2022). Although the VA is a comprehensive healthcare system that offers physical and mental health care along with various homeless services, respondents reported that some HEV still have access barriers; thus, providing outreach and onsite services where homeless Veterans reside may increase engagement (Tsai et al. 2022). Consistent with these findings, national dissemination studies emphasize that flexible, low‐demand, and outreach‐driven service models markedly improve engagement and housing retention among Veterans (Kinney et al. 2025). Similarly, the present study observed that rigid eligibility criteria and staffing limitations continue to restrict service reach.

Employment and job training are essential to sustain Veterans' permanent housing. Job training initiatives can enhance Veterans' employability by aligning their skills with market demands (Elbogen et al. 2023). These approaches promote self‐sufficiency and foster a sense of purpose and belonging within communities (Katie E. Davenport et al. 2023). Stable employment has also been associated with reduced risk of housing instability among Veterans, particularly when paired with supportive services and individualized vocational planning(Tsai and Rosenheck 2015). The VA offers employment programs like the Homeless Veterans Community Employment Services and Veteran Readiness and Employment; but challenges remain in accessibility, coordination, and long‐term job retention. Veterans face barriers such as limited access, employer stigma, and training that do not align with local job markets. To enhance effectiveness, better integration with housing and healthcare, tailored regional training, and stronger employer partnerships are essential (U. S. Department of Veteran Affairs 2024b).

Lastly, our study findings demonstrate the need for continued and strengthened collaborations between the VA and community organizations to mitigate Veteran homelessness. The VA homeless programs office collaborates with over 30 Community Resource and Referral Centers located in mostly large urban areas. These supportive services are critical to helping HEV find and retain housing. For HEV, these services serve as a platform for achieving health, recovery, and economic success. The 2024 VA Homeless Programs Office Report emphasizes cross‐sector collaboration as mandated by the U.S. Department of Veteran Affairs (2019), reinforcing that sustained progress depends on aligning federal and local partnerships with front‐line provider feedback (Allegrante and Sleet 2021; U. S. Government Publishing Office 2020; U.S. Department of Veteran Affairs 2019). There continues to be an ongoing need for collaborations with federal and community stakeholders to ensure Veterans have access to quality supportive health, mental health, and legal services alongside employment and housing assistance provided by the VA (U. S. Department of Housing and Urban Development 2021).

This study had some limitations. It was cross‐sectional in design, so it is hard to determine whether the need for specific resources and services to mitigate Veteran homelessness may vary over time. Hence, longitudinal data are needed to determine whether these responses are stable or have improved over time. The respondents in this study represent a convenience sample that may be subject to bias and non‐representative of the total HEV population, therefore limiting the generalizability of the findings. The data collected were self‐reported; therefore, the most important need(s) may vary by perceptions, lived experience, and the extent to which respondents choose to report their opinions. Because these findings derive from a single open‐ended question within the CHALENG survey, they represent participants' perceived priorities rather than an exhaustive list of needs. Although these limitations must be considered, the study's strength lies in its use of a heterogeneous national sample of HEV and VA providers, offering actionable insights into resource needs.

## 13 Conclusion

Despite significant progress, ending Veteran homelessness remains a national priority (U. S. Department of Housing and Urban Development 2023a, 2023b). In this study, providers and HEV shared their perceptions of resources and service gaps that continue to hinder efforts to mitigate homelessness. They emphasized the need to expand affordable, safe housing options, improve access to comprehensive treatment and supportive services, enhance educational opportunities, and strengthen collaboration across community, local, and state levels. Because this analysis draws on responses to a single open‐ended survey question, the findings should be interpreted as descriptive and exploratory, offering insight into perceived priorities rather than exhaustive needs. Future research could enhance the utility of CHALENG data by developing methods to securely link survey results with administrative or programmatic data sources, enabling analysis of trends in needs over time. Addressing the anonymous, cross‐sectional design would also clarify whether the same participants contribute to multiple survey cycles and strengthen the ability to assess changes in perceived resource needs. In addition, CHALENG results can inform both local and national efforts to strengthen coordination between VA and community partners and to monitor the impact of ongoing initiatives. This reinforces CHALENG's role as a mechanism for continuous learning, collaboration, and accountability in the collective effort to end Veteran homelessness.

## Author Contributions

Contributions: Lourence Misedah‐Robinson and Rebecca. L. Kinney were responsible for all stages of the article development including data analysis, interpretation, and drafting the manuscript. Jack Tsai was involved in supervision and resources and reviewed and edited manuscript drafts. All three authors approved the final manuscript draft.

## Patient Consent Statement

This quality‐improvement project was deemed exempt from oversight by the appropriate ethical review boards, as the VA Homeless Program Office implements the CHALENG survey as part of quality improvement. The project was deemed exempt from oversight with approval from the Office of Management and Budget (OMB). Thus, no patient consent was required.

## Conflicts of Interest

The authors declare no conflicts of interest.

## Data Availability

The data supporting this study's findings are available from the corresponding author, LMR, upon reasonable request. The data that support the findings of this study are available on request from the corresponding author. The data are not publicly available due to privacy or ethical restrictions.
